# Trajectories of perioperative serum CEA and non-small cell lung cancer prognosis: a retrospective longitudinal cohort study

**DOI:** 10.3389/fonc.2025.1627122

**Published:** 2025-10-14

**Authors:** Yuyang Ma, Xiaoyin Pan, Jiameng Cui, Wanzhu Lu, Gege Sun, Junlong Pan, Xiao Dong, Kejia Hu, Wenyuan Li, Huakang Tu, Xifeng Wu

**Affiliations:** ^1^ Department of Big Data in Health Science School of Public Health, Center of Clinical Big Data and Analytics of The Second Affiliated Hospital, Zhejiang University School of Medicine, Hangzhou, China; ^2^ National Institute for Data Science in Health and Medicine, Zhejiang University, Hangzhou, Zhejiang, China; ^3^ School of Medicine and Health Science, George Washington University, Washington, DC, United States; ^4^ Zhejiang Key Laboratory of Intelligent Preventive Medicine, Hangzhou, Zhejiang, China

**Keywords:** non-small lung cancer, curative resection, carcinoembryonic antigen, perioperative period, trajectories

## Abstract

**Background:**

The role of postoperative carcinoembryonic antigen (CEA) levels in non-small lung cancer (NSCLC) prognostic evaluation remains unclear. Additionally, the dynamic changes in CEA levels during the perioperative period have not been fully characterized.

**Methods:**

We retrospectively reviewed stage I-IIIA NSCLC patients who underwent curative resection. A latent class growth mixed model was employed to categorize patients into distinct CEA trajectory groups. The Kaplan-Meier method assessed the relationship between CEA trajectory groups and recurrence-free survival (RFS) and overall survival (OS). Multivariate analysis of perioperative CEA levels in relation to RFS and overall survival OS was performed using Cox proportional hazards regression.

**Results:**

A total of 5733 patients were included in our study. Elevated postoperative CEA levels were associated with higher risks of recurrence (HR = 2.64, 95% CI: 1.65-4.23) and mortality (HR = 3.34, 95% CI: 2.09-5.80) compared to normal CEA levels. Furthermore, patients with normal preoperative CEA but elevated postoperative levels also had higher risks of recurrence (HR = 3.00, 95% CI: 1.77–5.10) and mortality (HR = 3.30, 95% CI: 1.79–6.07). Three CEA trajectory categories were identified: low-stable, early-rising, and later-rising. Compared to the low-stable group, the early-rising group had significantly higher risks of recurrence (HR = 10.84, 95% CI: 5.57-21.10) and mortality (HR = 13.37, 95% CI: 5.45-32.81). The later-rising group had lower, but still significant, risks of recurrence (HR = 3.56, 95% CI: 1.62-7.81).

**Conclusion:**

Continuous postoperative monitoring of CEA levels in NSCLC patients is essential, especially for those with elevated postoperative CEA levels.

## Introduction

On a global scale, lung cancer continues to rank first in both new cancer cases and cancer - related fatalities (1). Among them, NSCLC accounts for around 80% to 85% of all lung cancer instances (2, 3). For patients diagnosed with localized non-small cell lung cancer (stage I-IIIA), the primary therapeutic approach is radical resectional operation. Unfortunately, recurrence-free survival (RFS) decreased from 68% at 1 year to 34% at 5 years for stage III, significantly reducing long-term survival rates (4). Therefore, early identification of patients with poor prognosis has become a key focus in the postoperative management of NSCLC.

CEA, a glycoprotein that contributes to the process of cell adhesion, is widely recognized as a crucial indicator for NSCLC (5, 6). Numerous meta-analyses have established a clear link between high preoperative CEA levels and reduced survival in NSCLC patients (7–9). However, whether the levels of carcinoembryonic antigen (CEA) measured after the operation can accurately predict prognosis remains a matter of debate. While some studies have pinpointed that increased CEA values post-surgery act as a factor indicating a negative outcome for those suffering from NSCLC others have not detected a substantial relationship (10–16). The majority of those research works focused on patients at the initial disease stage and employed different threshold values for elevated CEA. Additionally, the dynamic changes in serum CEA levels following surgery have often been overlooked, and the perioperative CEA trajectory has not been well defined. Consequently, the relationship between these trajectories and NSCLC outcomes is still unclear.

The objective of this study was to assess the role of postoperative serum CEA as a prognostic indicator in NSCLC and to determine whether changes in perioperative CEA levels provide additional insight into patient outcomes. These dynamic changes include variations from preoperative to postoperative CEA levels and the trajectories of CEA changes from preoperative to 36 months post-surgery. Additionally, we examined how pre- and postoperative CEA concentrations, treated as continuous variables, were associated with clinical outcomes in NSCLC patients.

## Methods

### Study framework and data acquisition

The study, designed as a retrospective cohort analysis, received ethical approval from the Institutional Review Board of the Second Affiliated Hospital of Zhejiang University School of Medicine (2020LSYD829). Patients with stage I to IIIA NSCLC who received curative surgery at the Second Affiliated Hospital between June 2015 and September 2023 were consecutively recruited. The following were the criteria for participants to be included in this research: 1) Histologically confirmed diagnosis of primary NSCLC. 2) No history of other cancers. 3) No local surgical treatments other than radical resection. 4) Negative surgical margins with no residual lesions. 5) No neoadjuvant therapy.

Serum CEA concentrations were measured via chemiluminescent immunoassay using the COBAS 8000 e602 analyzer (Roche Diagnostics, Tokyo, Japan), in accordance with the World Health Organization (WHO) international standard 73/601 (17). The pre-surgical CEA level was defined as the concentration measured within 30 days prior to resection. Patients with preoperative measurements were included in preoperative -related studies (**Supplementary Figure S1a**). The postoperative CEA level referred to the measurement closest to the surgical date, taken within seven months after resection. Patients with postoperative measurements were included in postoperative-related studies (**Supplementary Figure S1b**). For trajectory analysis, patients who had CEA measurements taken before the operation and whose CEA levels were measured at least three times during the 36-month period following the surgery were enrolled. All included CEA measurements after surgery were taken before any clinical outcomes occurred.

The study’s follow-up was completed on November 2, 2023. Recurrence-free survival served as the primary outcome, capturing both local and metastatic disease events. The secondary endpoint focused on overall survival (OS). Recurrence-free duration was calculated from the date of surgery to the time of relapse detection. which was established through histological examination of biopsy specimens or positive findings on imaging studies. Survival information was obtained through telephone follow-ups.

Covariates included age, sex, smoking status (yes or no), surgical methods (wedge resection, segmentectomy, or lobectomy), pathological stage (I-IIIA), histological subtype (adenocarcinoma or squamous cell carcinoma), and tumor differentiation grade (highly - matured, intermediately - matured, or poorly - matured). Preoperative CEA levels were also accounted for in the analysis of the relationships between trajectories and NSCLC outcomes. Data were retrieved from internal departmental records and digital medical archives. The pathological staging was assigned based on the 8th edition of the American Joint Committee on Cancer (AJCC) classification criteria for lung malignancies.

### Statistical analysis

**Figure 1:**
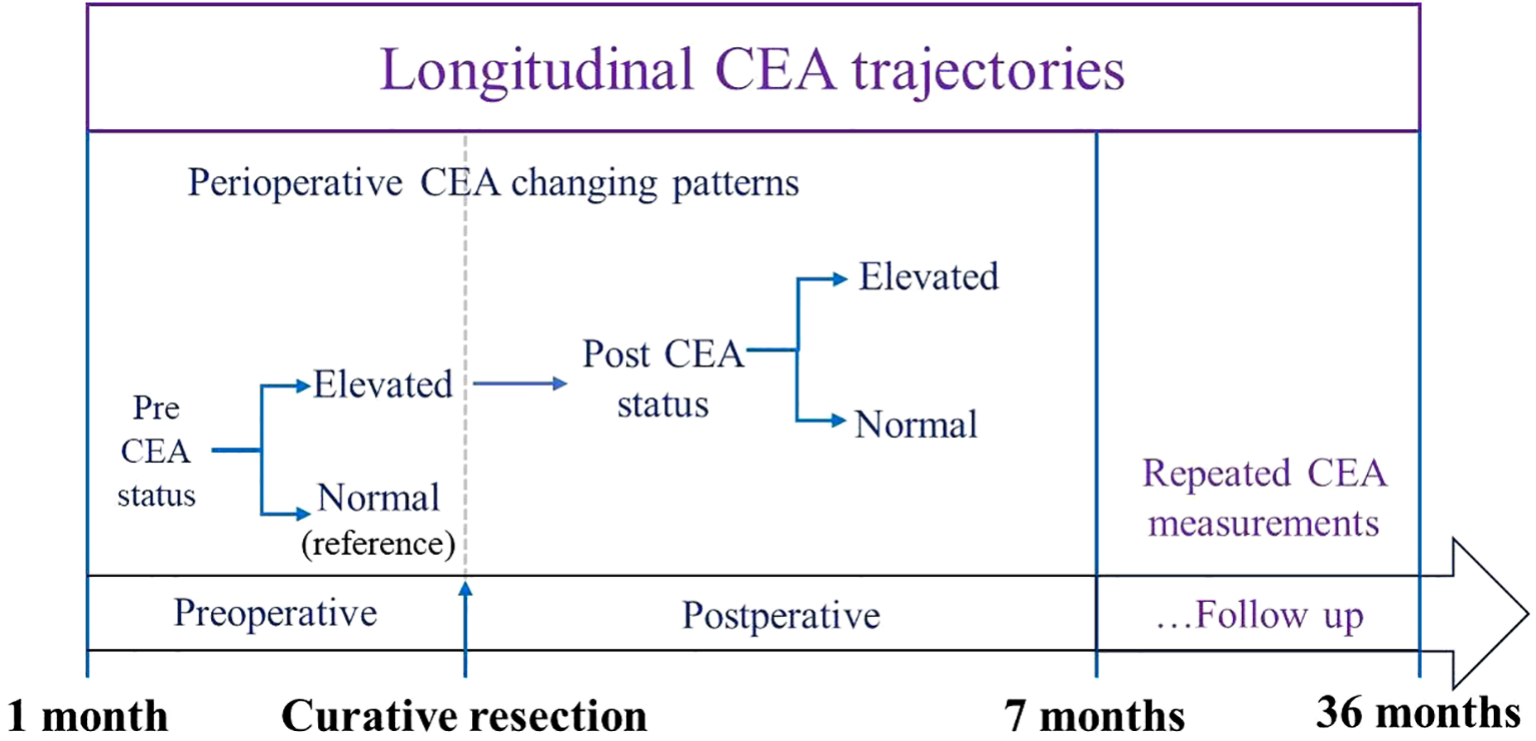
Workflow of CEA trajectory analysis: from preoperative baseline to 36-month postoperative follow-up.

The study workflow is depicted in the diagram shown in **Figure 1**. All the statistical analyses were conducted in R (v4.4.2), with missing values addressed through multiple imputation using the “mice” package (v2.1.0). We looked into the differences between groups. For continuous variables (stated as median [interquartile range]), the Kruskal - Wallis test was replaced with a non - parametric bootstrap - based test to assess group differences. Categorical data (presented as n [%]) were compared using the G-test, which serves as a likelihood-ratio alternative to the classical chi-square test.

CEA was classified as elevated when exceeding 5 ng/mL, and normal when at or below this threshold, according to standard reference values. Combining preoperative and postoperative levels further categorized into normal preoperative, normalization, and persistently elevated groups. Given the substantial variability within CEA measurements, values were capped at tenfold the upper reference limit to improve trajectory fitting. The logarithms of these truncated values were subsequently employed to model trajectories, due to the fact that they exhibited a non-normal distribution.

The application of a latent class growth mixed model (LCGMM) enabled the identification of distinct trajectory patterns of perioperative CEA levels. This method segments the population into latent classes based on estimated heterogeneity and constructs individual trajectories using a linear mixed model framework (18, 19). We modeled the long - term variations in the measured values as either linear or non - linear relationships with respect to the passage of time. These relationships included time, its second - order or third - order terms. We explored 2 to 5 potential trajectory groups. The number of optimal clusters and the most suitable curve form were selected based on the Bayesian Information Criterion (BIC), with the constraint that each class represented ≥ep of the cohort and exhibited an average posterior probability above 0.7. The “he.eb package (version 1.9.2) was used to perform the latent class growth mixture modeling. Kaplan-Meier curves were generated for overall survival (OS) and recurrence-free survival (RFS), with intergroup differences evaluated using the log-rank test. Associations between trajectory classes and outcomes were further analyzed using Cox regression models. The proportional hazards assumption was assessed using log-log plot. We made use of three models: the first was unadjusted; the second controlled for age, sex, and smoking status; and the third further accounted for surgical type, pathological stage, histological subtype, and tumor differentiation. In analyses involving CEA trajectory groups, preoperative CEA levels were also taken into account. We also constructed three more statistical models incorporating preoperative CEA, postoperative CEA, and both, respectively. Model comparisons were conducted via likelihood ratio tests (LRT). To flexibly characterize nonlinear effects, Restricted cubic splines (RCS) modeling was applied to assess how continuous pre- and postoperative CEA levels relate to recurrence risk in NSCLC. In survival analysis, RCS can model nonlinear relationships in Cox proportional hazards regression models (20, 21). Three knots were selected when modeling. The model took into account factors such as patient’s age, gender, smoking status, types of surgical procedures, disease stage based on pathology reports, pathological characteristics, and the extent of cellular differentiation.

## Results

### Study participants

**Figure 2:**
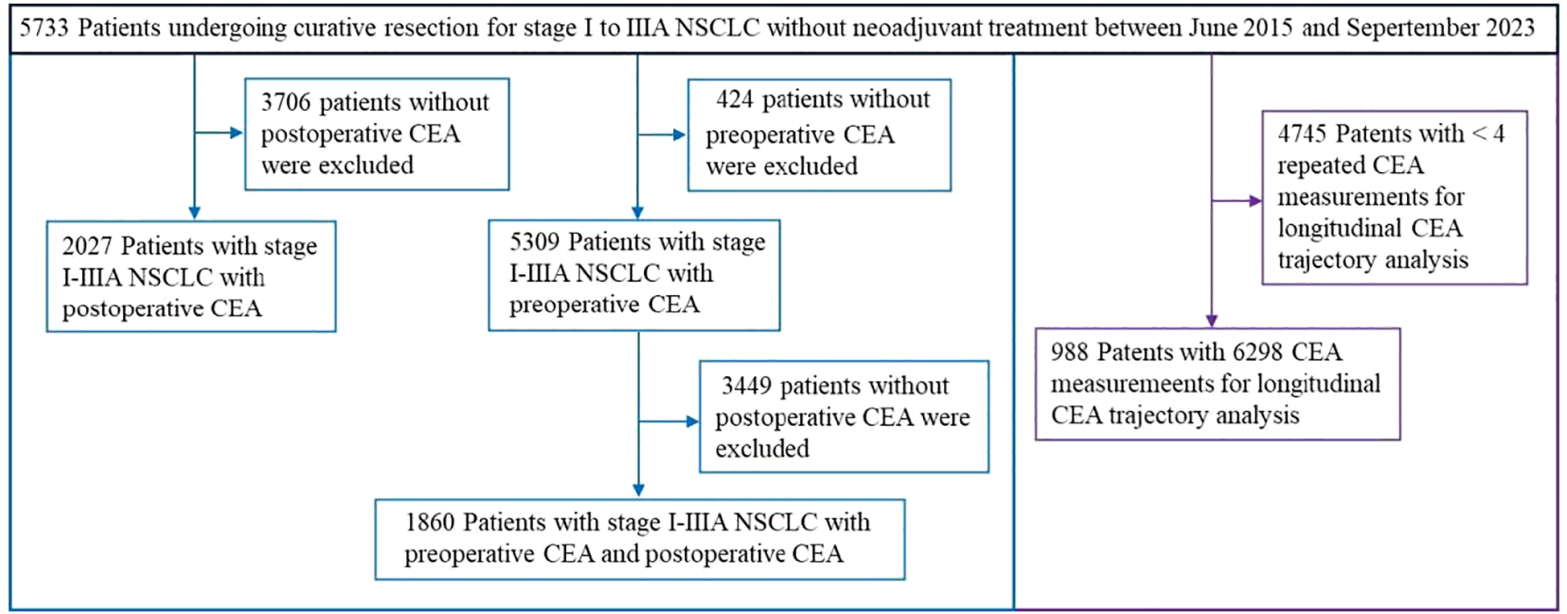
Flowchart of patient selection for longitudinal CEA monitoring in curatively resected NSCLC.

The study included 5733 individuals diagnosed with NSCLC. To elaborate, 5309 were incorporated into the preoperative analysis, 2027 into the postoperative investigation, 1860 into the study examining dynamic changes in the perioperative, periods and 988 into the CEA trajectory analysis. A summary of participant evaluation and exclusion rationale can be found in **Figure 2**.

### Trajectory analysis of CEA

**Figure 3:**
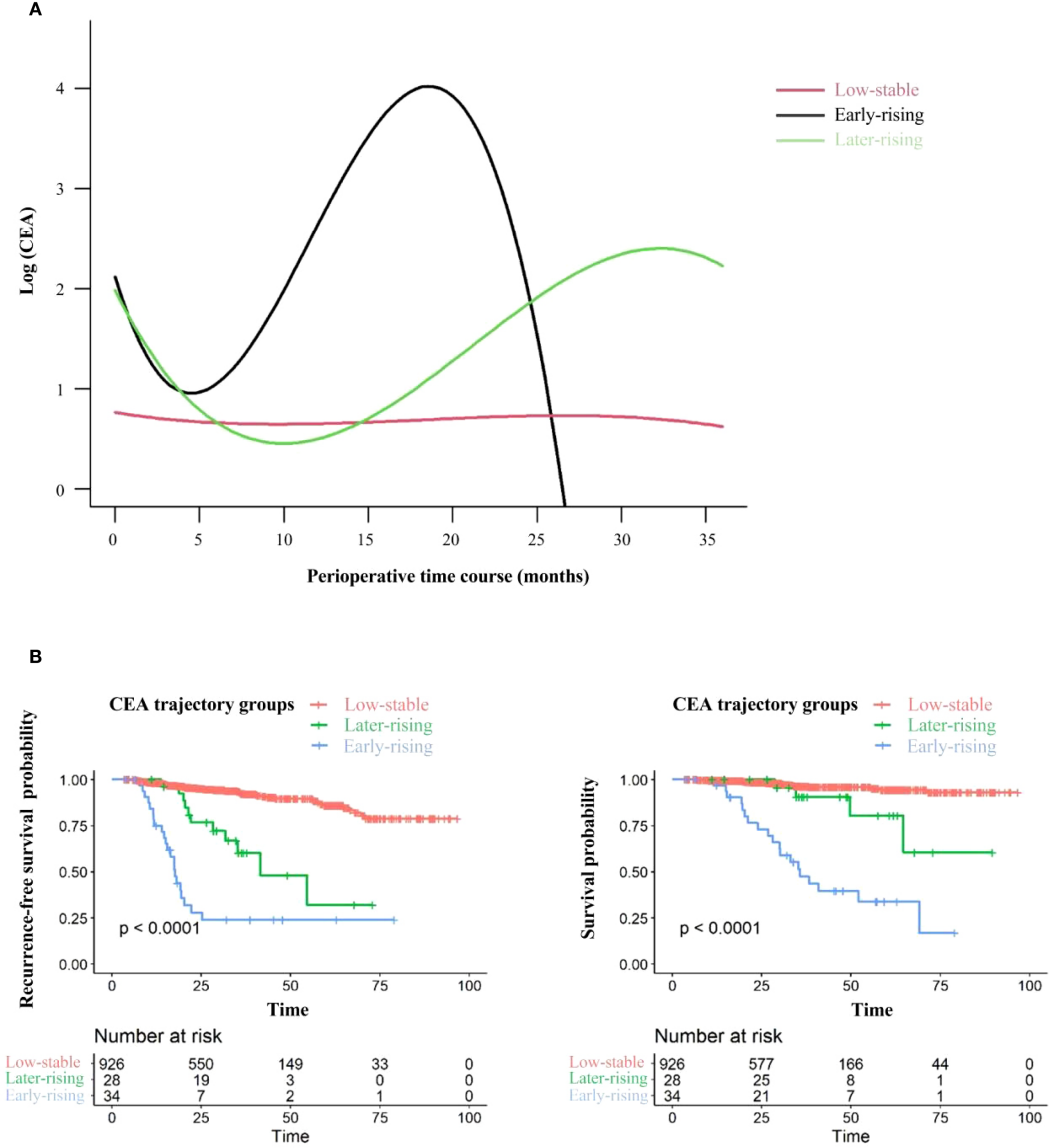
CEA trajectories and related recurrence-free survival (RFS) and overall survival outcomes. **(A)** CEA trajectories from the preoperative period to 36 months post-surgery. **(B)** Recurrence-free survival (left) and overall survival (right) stratified by CEA trajectory groups: comparisons between the Low-stable CEA group, Early-rising CEA group, and Later-rising CEA group.

The study included 988 patients (435 [44.03%] male; median age [interquartile range, IQR]: 60 years [52-67]). The median follow - up duration was 29.54 months (IQR: 18.33-38.21). Recurrence occurred in 98 patients (9.92%) during follow-up, yielding an incidence of 5.44 per 1,000 person-years. Meanwhile, 48 patients (4.86%) died, with a corresponding rate of 2.50 per 1,000 person-years. A total of 6298 individual CEA measurements were assessed, averaging a median of 6 measurements per patient (range: 4-36) (**Supplementary Figure S2**). The LCGMM model fitting results are presented in **Supplementary Table S1**. In line with the predefined guidelines, non - linear curves representing three possible clusters were considered the best fit for CEA modeling. **Figure 3A** depicts the perioperative trajectory of CEA concentration. Three separate trajectory groups were pinpointed for perioperative CEA: low-stable (n = 926, 93.72%), early-rising (n = 34, 3.44%), and later-rising (n = 38, 3.85%). Among the members of the low-stable group, CEA levels consistently remained below 5.0 ng/mL from the preoperative period through 36 months after surgery. In the early -rising group, CEA decreased to its lowest value at 5 months post-surgery, followed by a rapid increase. In the later - rising group, CEA dropped to the standard reference interval within 6 months post - surgery and gradually ascended after 10 months.

Relative to the group with consistently low CEA levels, the groups demonstrating an early increase and a late increase in CEA levels were characterized by lower tumor stages and differentiation levels. Additionally, fewer patients in these rising groups underwent wedge resection or segmentectomy. Both rising groups also demonstrated elevated risks of recurrence and mortality (**Table 1**). The 5-year RFS rates were 23.85% (95% CI: 12.10%–47.02%) for the early-rising group, 32.00% (95% CI: 12.00%–85.30%) for the later-rising group, and significantly higher at 86.15% (95% CI: 81.99%–90.52%) in the low-stable group (*P* < 0.001; **Figure 3B**, left). A similar trend was observed for overall survival: the 5-year OS rate reached 94.55% (95% CI: 92.00%–97.18%) in the low-stable group, compared to 33.93% (95% CI: 19.47%–59.13%) in the early-rising group and 80.50% (95% CI: 61.60%–100.00%) in the later-rising group (*P* < 0.001; **Figure 3B**, right). Sub-analysis stratified by histology shows that in adenocarcinoma patients, the patterns in 5-year RFS and OS across the three CEA trajectory groups are highly consistent with those observed in the overall population (**Supplementary Figures S5a, b**).

**Table 1:** Characteristics by serum CEA trajectory groups at baseline and follow-up.

Characteristic	Low-stable (n=926)	Later-rising (n=28)	Early-rising (n=34)	All (N = 988)	*P* value
Baseline					
Preoperative CEA, ng/ml	2.20 [1.50,3.50]	11.15 [5.38,30.45]	15.40 [8.10,32.17]	2.30 [1.50,3.80]	<0.001
Smoking, n (%)	289 (31.2)	9 (32.1)	15 (44.1)	313 (31.68)	0.310
Female, n (%)	519 (56.0)	18 (64.3)	16 (47.1)	553 (55.41)	0.390
Age, years	60.00 [52.00,67.00]	64.00 [56.00,67.25]	60.0 [55.25,68.75]	60.00 [52.00,67.00]	0.325
Degree					<0.001
well-differentiated	21 (2.3)	0 (0.0)	0 (0.0)	21 (2.1)	
moderately-differentiated	95 (10.3)	1 (3.6)	2 (23.5)	98 (9.92)	
poorly-differentiated	135 (14.6)	8 (28.6)	9 (76.5)	152 (15.38)	
AJCC 8^th^ ed.stage, n (%)					<0.001
I	773 (83.5)	10 (35.7)	7 (20.6)	790 (79.16)	
II	77 (8.3)	6 (21.4)	8 (23.5)	91 (9.12)	
III9	76 (8.2)	12 (42.9)	19 (55.9)	107 (10.72)	
Pathology, n (%)					0.469
squamous cell carcinoma	119 (12.9)	2 (7.1)	6 (17.6)	127 (12.73)	
adenocarcinoma	807 (81.1)	26 (92.9)	28 (82.4)	861 (87.27)	
Surgery methods, n (%)					<0.001
wedge resection	381 (41.1)	21 (75.0)	28 (82.4)	444 (44.49)	
segmentectomy	346 (37.4)	5 (17.9)	3 (9.4)	366 (36.67)	
lobectomy	161 (17.4)	0 (0.0)	2 (5.9)	178 (17.84)	
Follow-up					
death, n (%)	25 (2.7)	4 (14.3)	19 (55.9)	48 (4.81)	<0.001
recurrence, n (%)	65 (7.0)	11 (39.3)	22 (64.7)	98 (9.82)	<0.001

For continuous variables, data are presented as median [interquartile range]. For categorical variables, data are presented as count [%].

**Tables 2**, **3** summarize the relationships between perioperative CEA trajectories and both RFS and OS. Relative to the low-stable group, the risk of recurrence was significantly higher in the early- and late-rising groups, with hazard ratios (HRs) of 10.84 (95% CI: 5.57–21.10, *P* < 0.001) and 3.56 (95% CI: 1.62–7.81, *P* = 0.002), respectively. After adjustment for demographic characteristics and preoperative CEA, the associations were slightly attenuated but remained significant. The early-rising group showed a markedly higher risk of death (HR = 13.37, 95% CI: 5.45–32.81, *P* < 0.001) relative to the low-stable group, whereas the mortality risk for the later-rising group was not statistically significant (HR = 2.00, 95% CI: 0.58–6.93, *P* = 0.276) following full adjustment.

**Table 2:** Exploration of the relationship between perioperative and longitudinal CEA groups and recurrence based on Cox proportional hazards regression.

	n	recurrence, n (%)	Model 1		Model 2		Model 3	
			Hazard ratio (95%CI)	*P* value	Hazard ratio (95%CI)	*P* value	Hazard ratio (95%CI)	*P* value
Preoperative CEA status								
Preoperative CEA^*^	5309	198 (3.7%)	1.54 (1.41-1.67)	<0.001	1.51 (1.39-1.66)	<0.001	1.31 (1.19-1.46)	<0.001
normal preoperative	4739	128 (2.7%)	reference		reference		reference	
elevated preoperative	570	70 (12.3%)	3.98 (2.97-5.33)	<0.001	3.70 (2.70-4.93)	<0.001	2.49 (1.80-3.44)	<0.001
Postoperative CEA status								
Postoperative CEA^*^	2027	154 (7.6%)	1.69 (1.433-1.983)	<0.001	1.66 (1.39-1.96)	<0.001	1.53 (1.29-1.81)	<0.001
normal postoperative	1921	132 (6.9%)	reference		reference		reference	
elevated postoperative	106	22 (20.8%)	4.00 (2.55-6.28)	<0.001	3.68 (2.33-5.83)	<0.001	2.64 (1.65-4.23)	<0.001
Perioperative CEA status								
normal perioperative	1608	90 (5.6%)	reference		reference		reference	
normalized postoperative	170	29 (17.1%)	2.83 (1.86-4.30)	<0.001	2.64 (1.73-4.03)	<0.001	1.82 (1.17-2.85)	0.008
elevated postoperative	82	19 (23.2%)	5.05 (3.08-8.28)	<0.001	4.84 (2.93-8.00)	<0.001	3.00 (1.77-5.10)	<0.001
Longitudinal CEA trajectory groups during preoperative to 36 months after surgery								
low-stable	926	65 (7.0%)	reference		reference		reference	
later-rising	34	11 (39.3%)	5.57 (2.94-10.56)	<0.001	5.22 (2.44-11.18)	<0.001	3.56 (1.62-7.81)	0.002
early-rising	28	22 (64.7%)	15.97 (9.79-26.03)	<0.001	14.65 (7.80-27.52)	<0.001	10.84 (5.57-21.10)	<0.001

* denoted continuous, log2-transformed variables. Model 1 was unadjusted.

Model 2 was adjusted for age, sex(male vs. female) and smoking (yes vs. no).

Model 3 was adjusted for age, sex(male vs. female) and smoking (yes vs. no), pathology stage (I vs. II vs. IIIA), pathology (squamous cell carcinoma vs. adenocarcinoma), surgical methods (wedge resection vs. segmentectomy vs. lobectomy), and degree of differentiation(well-differentiated vs. moderately-differentiated vs. poorly-differentiated).

**Table 3:** Cox proportional hazards regression analysis assessing the effects of perioperative and longitudinal CEA groups on survival outcomes.

	n	death,n (%)	Model 1		Model 2		Model 3	
			Hazard ratio (95%CI)	*P* value	Hazard ratio (95%CI)	*P* value	Hazard ratio (95%CI)	*P* value
Preoperative CEA status								
Preoperative CEA^*^	5309	129 (2.4%)	1.59 (1.45-1.75)	<0.001	1.54 (1.39-1.71)	<0.001	1.24 (1.10-1.40)	<0.001
normal preoperative	4739	72 (1.5%)	reference		reference		reference	
elevated preoperative	570	57 (10.0%)	4.74 (3.34-6.72)	<0.001	3.90 (2.74-5.54)	<0.001	2.26 (1.54-3.33)	<0.001
Postoperative CEA status								
Postoperative CEA^*^	2027	85 (4.2%)	2.01 (1.71- 2.37)	<0.001	1.98 (1.65-2.37)	<0.001	1.72 (1.42-2.09)	<0.001
normal postoperative	1921	62 (3.2%)	reference		reference		reference	
elevated postoperative	106	23 (21.7%)	7.43 (4.60-12.00)	<0.001	6.53 (4.02-10.61)	<0.001	3.48 (2.09-5.80)	<0.001
Perioperative CEA status								
normal preoperative	1608	40 (2.5%)	reference		reference		reference	
normalized postoperative	170	19 (11.2%)	3.56 (2.06-6.16)	<0.001	3.23 (1.86-5.58)	<0.001	1.80 (1.01-3.22)	0.047
elevated postoperative	82	18 (22.0%)	8.63 (4.94-15.07)	<0.001	7.63 (4.34-13.41)	<0.001	3.30 (1.79-6.07)	<0.001
Longitudinal CEA trajectory groups during preoperative to 36 months after surgery								
low-stable	926	25 (2.7%)	reference		reference		reference	
later-rising	34	4 (14.3%)	3.83 (1.33-11.01)	0.013	3.46 (1.02-11.75)	0.046	2.00 (0.58-6.93)	0.276
early-rising	28	19 (55.9%)	20.65 (11.35-37.56)	<0.001	20.03 (8.73-45.93)	<0.001	13.37 (5.45-32.81)	<0.001

* denoted continuous, log2-transformed variables. Model 1 was unadjusted.

Model 2 was adjusted for age, sex(male vs. female) and smoking (yes vs. no).

Model 3 was adjusted for age, sex(male vs. female) and smoking (yes vs. no), pathology stage (I vs. II vs. IIIA), pathology (squamous cell carcinoma vs. adenocarcinoma), surgical methods (wedge resection vs. segmentectomy vs. lobectomy), and degree of differentiation(well-differentiated vs. moderately-differentiated vs. poorly-differentiated).

### Preoperative and postoperative analysis of CEA

**Figure 4:**
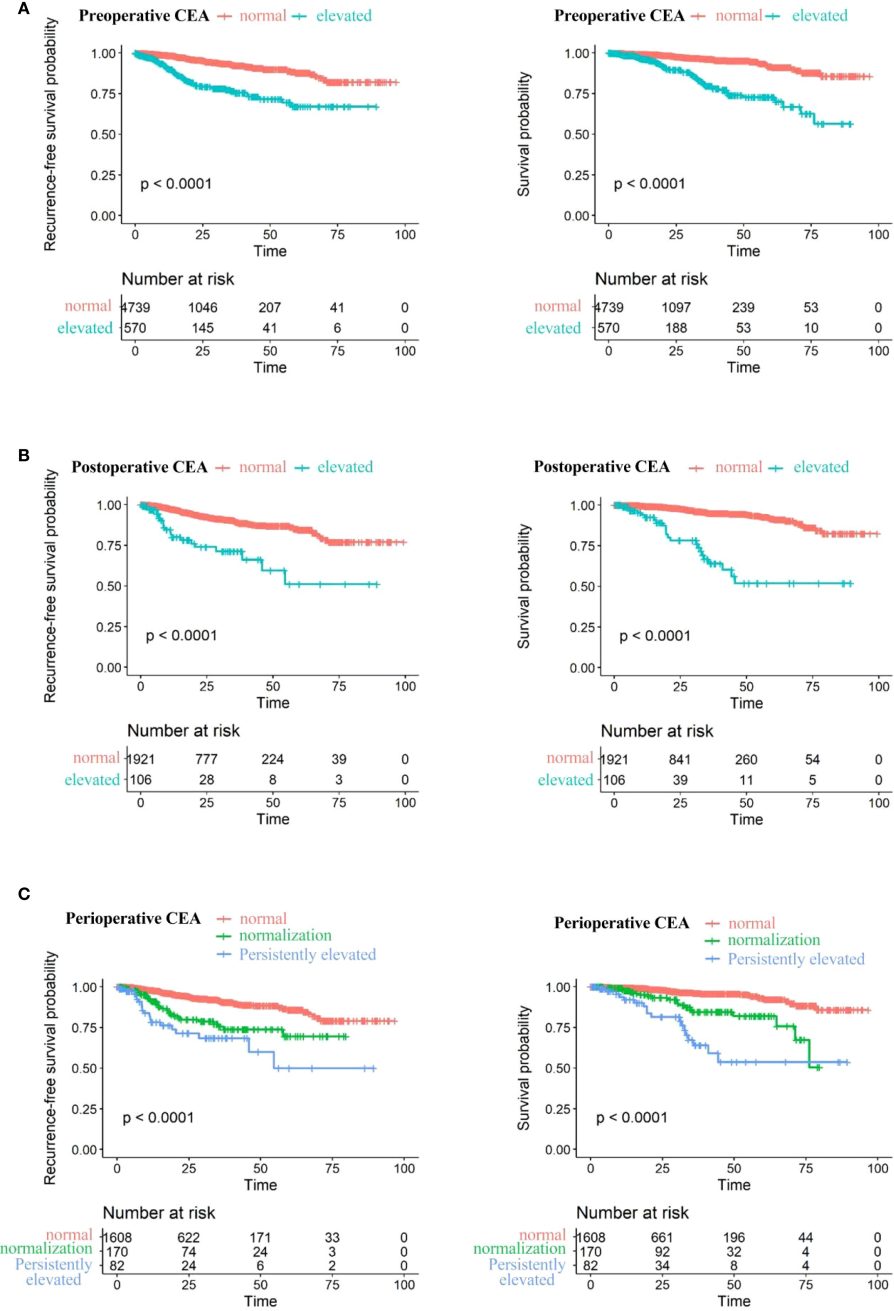
Recurrence-free survival (left) and overall survival (right) stratified by: **(A)** Preoperative CEA levels: normal (≤5 ng/ml) vs. elevated (>5 ng/ml). **(B)** stoperative CEA levels: normal (≤5 ng/ml) vs. elevated (>5 ng/ml). **(C)** Perioperative CEA levels: normal preoperative, elevated preoperative with postoperative normalization, and persistently elevated postoperatively.

**Figure 4** displays Kaplan-Meier survival curves stratified by preoperative, postoperative, and perioperative CEA profiles. The 5Cox model revealed that varying CEA levels were significantly associated with NSCLC prognosis, as shown in **Tables 2**, **3**. Higher preoperative log2-transformed CEA was associated with increased risk of recurrence (HR = 1.31, 95% CI: 1.19-1.46) and mortality (HR = 1.24, 95% CI: 1.10-1.40). Individuals with elevated preoperative CEA levels demonstrated significantly higher risks of recurrence (HR = 2.49, 95% CI:1.80-3.44) and mortality (HR = 2.26, (95% CI: 1.54-3.33) compared to those with normal preoperative CEA levels. Likewise, higher postoperative log2-transformed CEA was associated with increased risk of recurrence (HR = 1.53, 95% CI: 1.29-1.81) and mortality (HR = 1.72, 95% CI: 1.42-2.09). Individuals with elevated postoperative CEA levels demonstrated significantly higher risks of recurrence (HR = 2.64, 95% CI: 1.65-4.23) and mortality (HR = 3.34, 95% CI: 2.09-5.80) compared to those with normal postoperative CEA levels. After adjusting for preoperative CEA, postoperative CEA remained an independent predictor of mortality (HR = 2.13, 95% CI: 1.42–2.09), as shown in **Supplementary Table S2**.

Individuals with consistently elevated CEA levels both preoperatively and postoperatively exhibited markedly higher risks of recurrence and mortality, with HRs of 3.00 (95% CI: 1.77-5.10) for recurrence and 3.30 (95% CI: 1.79-6.07) for death. Patients whose CEA levels normalized after surgery still faced elevated risks of recurrence and death—HRs of 1.82 (95% CI: 1.17–2.85) and 1.80 (95% CI: 1.01–3.22), respectively—although these risks remained lower than in those with sustained CEA elevation.

### Restricted cubic splines analysis

**Figure 5:**
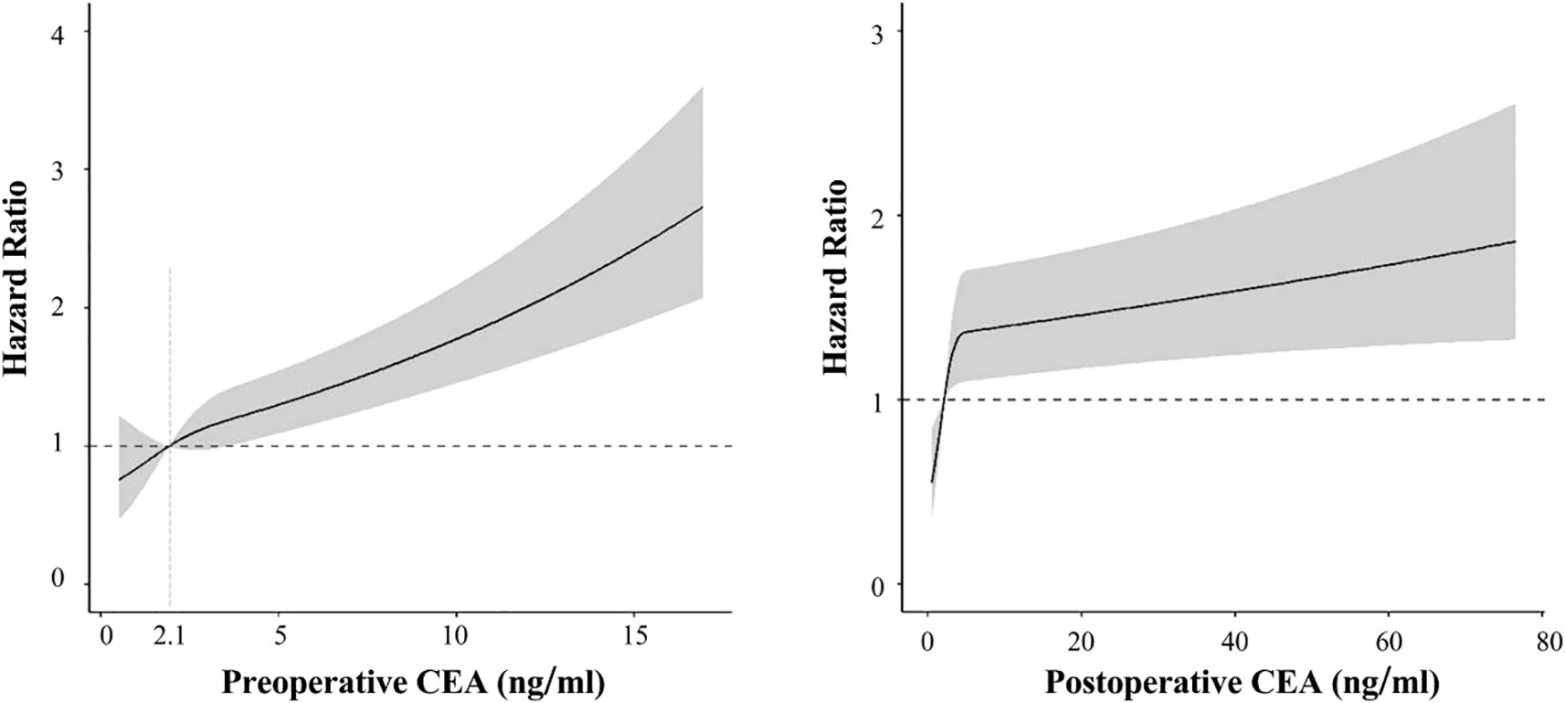
Association of preoperitive CEA (left) and postoperitive CEA (right) with recurrence-free survival in spline analysis.

Through multivariable-adjusted polynomial regression analysis (**Figure 5**), a positive nonlinear relationship was found between NSCLC recurrence risk and preoperative CEA levels (*P* for non-linearity=0.007). As preoperative CEA levels increased, so did the recurrence risk. The points where the hazard ratio for preoperative CEA levels equals 1.0 is approximately at 2.1 ng/mL. Below this threshold, the recurrence risk for NSCLC is less than 1.0, whereas above it, the recurrence risk exceeds 1.0. However, no non-linear association was detected between the levels of postoperitive CEA and the occurrence of recurrence (*P* for non-linearity =0.381).

## Discussion

Our study analyzed retrospective data from the SAH Lung Cancer Cohort and characterized three distinct longitudinal patterns of CEA dynamics spanning from the preoperative phase to extended follow-up: low-stable, early-rising, and late-rising. Compared to those with consistently low CEA levels, NSCLC patients with early or delayed increases faced elevated recurrence risks, with the early-rising group also exhibited a higher risks of death.

As research indicates that CEA levels in NSCLC patients typically return to normal within six weeks after surgery (22), our trajectory analysis revealed delayed CEA normalization (≤6 months) in the late-rising group and CEA levels reaching nadir at approximately 5 months in the early-rising group. This delay may reflect the current follow-up protocol, where most stage I-IIIA patients undergo CEA testing at 3–6 month intervals post-surgery. These results suggest that more frequent CEA monitoring could enable earlier detection of high-risk trajectories, particularly in patients with intraoperative findings suggestive of micrometastases such as visceral pleural invasion or lymphovascular invasion.

Our analysis indicated that the rising trend in CEA from preoperative to postoperative periods was closely related to poor prognosis, consistent with conclusions from other studies (16, 23). Persistently elevated postoperative CEA levels often suggest incomplete surgical resection or hidden metastasis, indicating cancer recurrence (14, 15). Even after adjusting for preoperative CEA levels and other clinicopathological covariates, the association between CEA trajectory groups and prognosis remained significant, suggesting that long-term CEA trajectories are independent prognostic factors for NSCLC recurrence and death. These trajectories likely reflect the tumor’s biological behavior, the effectiveness of surgical resection, adjuvant therapy, and the host’s immune defense over time, providing more prognostic information than preoperative CEA alone.

Our results are consistent with prior evidence, showing that heightened CEA concentrations before and after surgery are linked to poor outcomes in NSCLC. Notably, sustained elevation is strongly associated with increased recurrence and mortality risks. NSCLC patients whose preoperative CEA levels were elevated but returned to normal postoperatively still showed increased likelihood of disease relapse and mortality, though to a lesser extent than those with persistently high CEA levels. Therefore, a postoperative decrease in CEA to the normal range is a protective factor for NSCLC prognosis compared to levels remaining elevated postoperatively (24, 25).

A major advantage of this study is the analysis of discrete and irregular longitudinal CEA data collected during postoperative follow-up from a sizable patient cohort. This can help clinicians develop individualized follow-up strategies by observing changes in patients’ CEA trajectories. Although different immunoassay methods may be used to measure CEA, the normal range for CEA is consistent across methods (0–5 ng/mL), suggesting minimal impact of measurement differences on CEA values in future clinical practice.

However, there are limitations to our study. Firstly, we did not control for confounding factors such as comorbidities and postoperative adjuvant therapy, which could influence patient prognosis, although the number of patients receiving postoperative adjuvant therapy was relatively small (<5%). Thus, Renal failure can cause false-positive elevations in tumor markers, potentially affecting the results. Secondly, subgroup sizes for early- and late-rising CEA patterns were limited by their low occurrence rates. Additionally, as the cohort was confined to a single institution -Zhejiang Second Lung Cancer Cohort – this may limit the representativeness of the findings. Further investigation is warranted to assess the applicability of these findings to a more diverse lung cancer population. The long time span for postoperative CEA measurement (within seven months) could also affect the results. Restricting this to three months might provide more rigorous data. Furthermore, the exclusion of patients who underwent neoadjuvant therapy may limit the generalizability of the findings. Future studies will investigate the impact of serum CEA trajectories after neoadjuvant therapy on prognosis. Lastly, our study was retrospective, and its conclusions need validation in prospective cohorts or clinical trials.

In summary, our findings highlight the prognostic relevance of perioperative serum CEA dynamics in resected lung cancer patients. The results indicate that postoperative CEA trends may inform personalized surveillance strategies and provide clinically meaningful insights to support post-surgical management.

## Conclusion

CEA serves as a strong prognostic indicator for patients with NSCLC. Sustained elevations, whether observed preoperatively, postoperatively, or both, are linked to heightened risks of disease recurrence and mortality. Patients exhibiting persistently elevated CEA levels around the time of surgery are particularly vulnerable compared to those maintaining normal values throughout.

Our analysis identifies three distinct perioperative CEA trajectory groups in NSCLC patients: early-rising, late-rising, and stable. Both early- and late-rising groups exhibit higher recurrence rates compared to the stable group, with the early-rising group demonstrating the highest risk of mortality. This study underscores the critical importance of ongoing postoperative monitoring of CEA levels in NSCLC patients. Close surveillance is particularly warranted for those with rising CEA levels after surgery.

## Data Availability

The datasets generated and analyzed during this study are not publicly available due to restrictions imposed by the hospital’s data protection policy. Requests for data access may be directed to the corresponding author. Requests to access these datasets should be directed to XW, xifengw@zju.edu.cn.
