# Supplementary material for: Trajectories of perioperative serum CEA and non-small cell lung cancer prognosis: a retrospective longitudinal cohort study
Source: Front Oncol. 2025 Oct 14;15:1627122. doi: 10.3389/fonc.2025.1627122 (PMC12558766; doi:10.3389/fonc.2025.1627122)
Supplement: Supplementary file 1 [file DataSheet1.docx]

**Table S1. Latent class growth models (LCGMM) results of CEA model fitting process**

| No. Latent class | Polynomial degree | Log-Lik | BIC | % Participants per class | Mean posterior probabilities | % Posterior probabilities>70% |
| --- | --- | --- | --- | --- | --- | --- |
| 1 | Linear | -4270.149 | 8568 | 100 | NA | NA |
| 1 | Quadratic | -4233.944 | 8502 | 100 | NA | NA |
| 1 | Cubic | -4186.91 | 8415 | 100 | NA | NA |
| 2 | Linear | -3998.508 | 8045 | 3.34/96.66 | 0.91/0.99 | 84.85/99.37 |
| 2 | Quadratic | -3951.937 | 7966 | 96.25/3.75 | 0.99/0.92 | 99.79/86.49 |
| 2 | Cubic | -3771.062 | 7618 | 3.65/96.35 | 0.96/0.99 | 97.22/99.79 |
| 3 | Linear | -3930.733 | 7930 | 3.34/94.63/2.03 | 0.90/0.98/0.91 | 81.82/98.82/90.00 |
| 3 | Quadratic | -3729.024 | 7548 | 94.12/3.24/2.63 | 0.98/0.86/0.93 | 99.03/81.25/84.62 |
| **3** | Cubic | **-3523.489** | **7157** | **3.44/93.72/2.84** | **0.95/0.99/0.93** | **94.12/99.46/89.29** |
| 4 | Linear | -3865.907 | 7821 | 2.13/2.13/93.52/2.23 | 0.89/0.88/0.98/0.84 | 85.71/85.71/98.70/77.27 |
| 4 | Quadratic | -3648.367 | 7414 | 3.04/3.04/1.93/3.65 | 0.93/0.98/0.86/0.87 | 93.33/99.22/73.68/80.56 |
| 4 | Cubic | -3439.966 | 7025 | 91.89/91.89/2.74/1.72 | 0.99/0.90/0.93/0.87 | 99.56/86.11/92.59/88.24 |
| 5 | Linear | -3865.907 | 7842 | 0.00/2.53/92.81/2.23/2.43 | 0.37/0.81/0.61/0.86/0.81 | NA/72/0/81.82/70.83 |
| 5 | Quadratic | -3418.586 | 6982 | 2.33/2.33/90.58/1.82/2.94 | 0.95/0.92/0.98/0.89/0.88 | 91.30/91.30/98.88/83.33/86.21 |
| 5 | Cubic | -3178.791 | 6537 | 90.68/90.68/3.24/1.42/1.82 | 0.99/0.92/0.91/0.96/0.92 | 98.99/92.86/87.50/92.86/94.44 |

Note: No. Latent class: latent class number of the model; Log-Lik: the maximum Log-Likelihood; BIC: the Bayesian information Criterion; % Participants per class: proportion of participants per class; The best fitting model is highlighted in bold characters. NA: not applicable.

**Table S2. Cox proportional hazards regression analysis assessing the effects of perioperative and longitudinal CEA groups on survival outcomes.**

| Model | Variable | n | Outcome,  n(%) | Coefficient | hazard ratio (95%CI) | *p* value |
| --- | --- | --- | --- | --- | --- | --- |
| *Recurrence* | | | | | | |
| Model 1 | Preoperative CEA | 1860 | 138 (7.4%) | 0.77 | 2.14 (1.47-3.15) | <0.001 |
| Model 2 | Postoperative CEA | 1860 | 138 (7.4%) | 0.83 | 2.30 (1.39-3.83) | <0.001 |
| Modle 3 | Preoperative CEA | 1860 | 138 (7.4%) | 0.63 | 1.89 (1.22-2.91) | 0.004 |
|  | Postoperative CEA | 1860 | 138 (7.4%) | 0.42 | 1.52 ( 0.86-2.69) | 0.152 |
| *Mortality* | | | | | | |
| Model 1 | Preoperative CEA | 1860 | 77 (4.1%) | 0.83 | 2.28 (1.40-3.74) | 0.001 |
| Model 2 | Postoperative CEA | 1860 | 77 (4.1%) | 1.08 | 2.94 (1.70-5.09) | <0.001 |
| Modle 3 | Preoperative CEA | 1860 | 77 (4.1%) | 0.53 | 1.69 (0.95-3.03) | 0.077 |
|  | Postoperative CEA | 1860 | 77 (4.1%) | 0.76 | 2.13 (1.12-4.03) | 0.021 |

Note: Model 1 included preoperative CEA.
Model 2 included postoperative CEA.
Model 3 included both preoperative and postoperative CEA.
The covariates included in all models were age, sex (male vs. female), smoking status (yes vs. no), pathology stage (I vs. II vs. IIIA), histology (squamous cell carcinoma vs. adenocarcinoma), surgical method (wedge resection vs. segmentectomy vs. lobectomy), and degree of differentiation (well-differentiated vs. moderately differentiated vs. poorly differentiated).

**Table S3. Likelihood Ratio Test for Model Comparison of Recurrence and Mortality.**

| Model compared | LRT1 | Chisq Df | *p* value |
| --- | --- | --- | --- |
| *Recurrence* |  |  |  |
| Model 1 vs Model 3 | 1.96 | 1 | 0.161 |
| Model 2 vs Model 3 | 7.58 | 1 | 0.005 |
| *Mortality* |  |  |  |
| Model 1 vs Model 3 | 5.24 | 1 | 0.022 |
| Model 2 vs Model 3 | 2.99 | 1 | 0.084 |

Note: LRT^1^ represented the chi-square test statistic for the likelihood-ratio test.

Model 1 included preoperative CEA.
Model 2 included postoperative CEA.
Model 3 included both preoperative and postoperative CEA.
The covariates included in all models were age, sex (male vs. female), smoking status (yes vs. no), pathology stage (I vs. II vs. IIIA), histology (squamous cell carcinoma vs. adenocarcinoma), surgical method (wedge resection vs. segmentectomy vs. lobectomy), and degree of differentiation (well-differentiated vs. moderately differentiated vs. poorly differentiated).

**
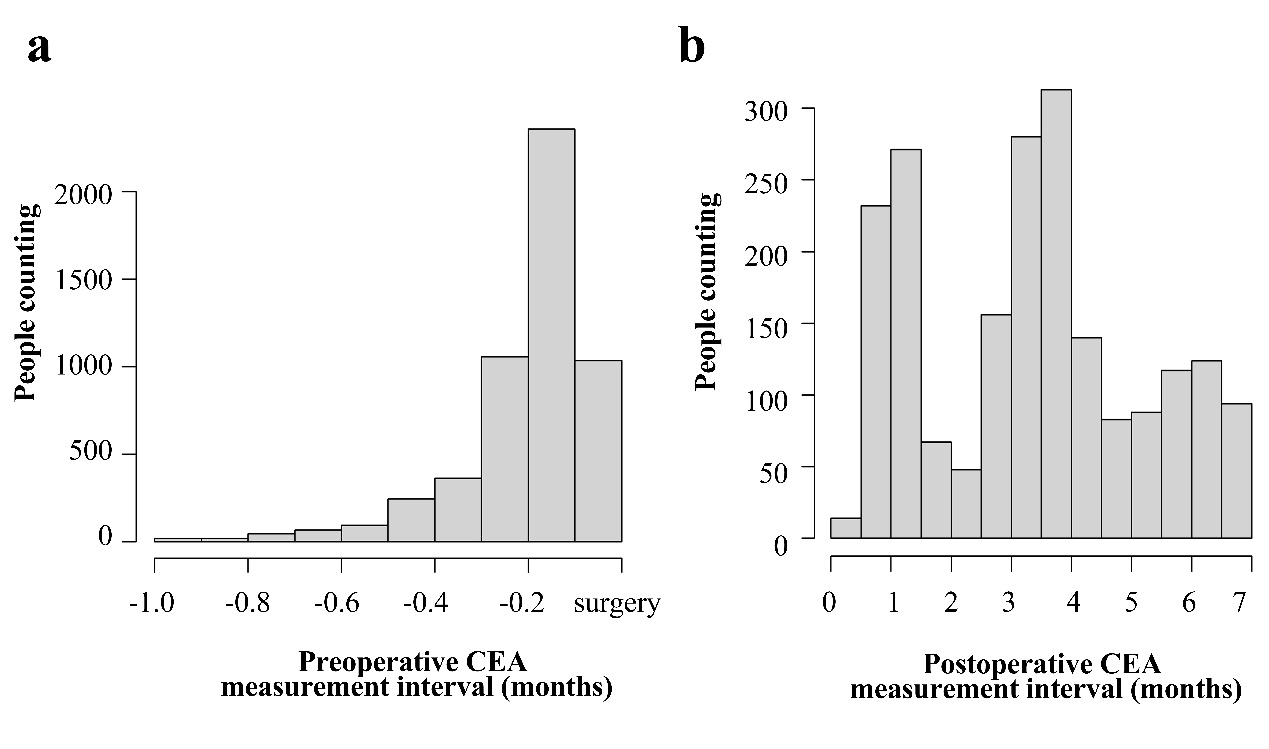
**

**Figure S1. Distribution of preoperative and postoperative CEA measurement intervals**


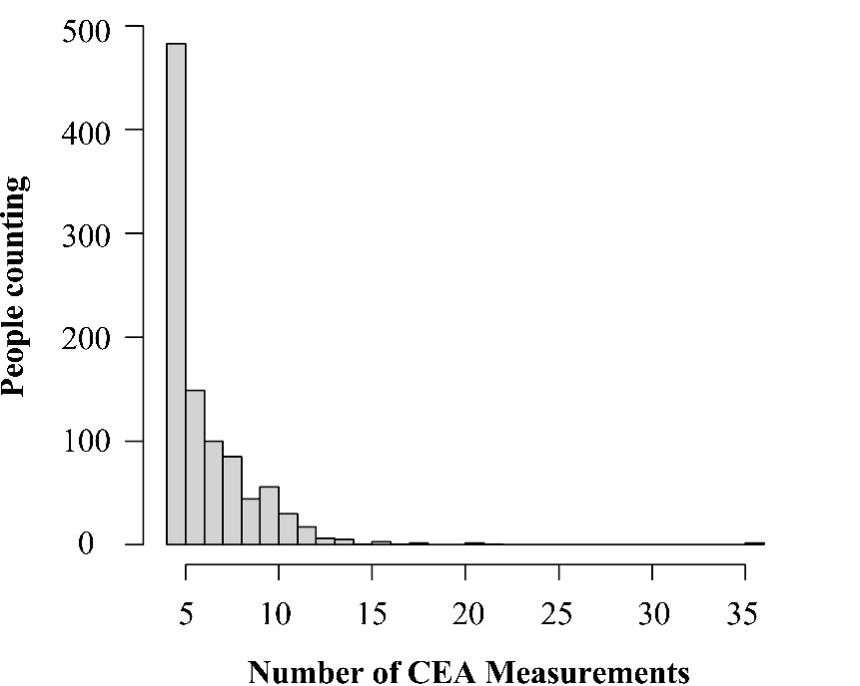


**Figure S2. Distribution of longitudinal CEA measurements in trajectory analysis**


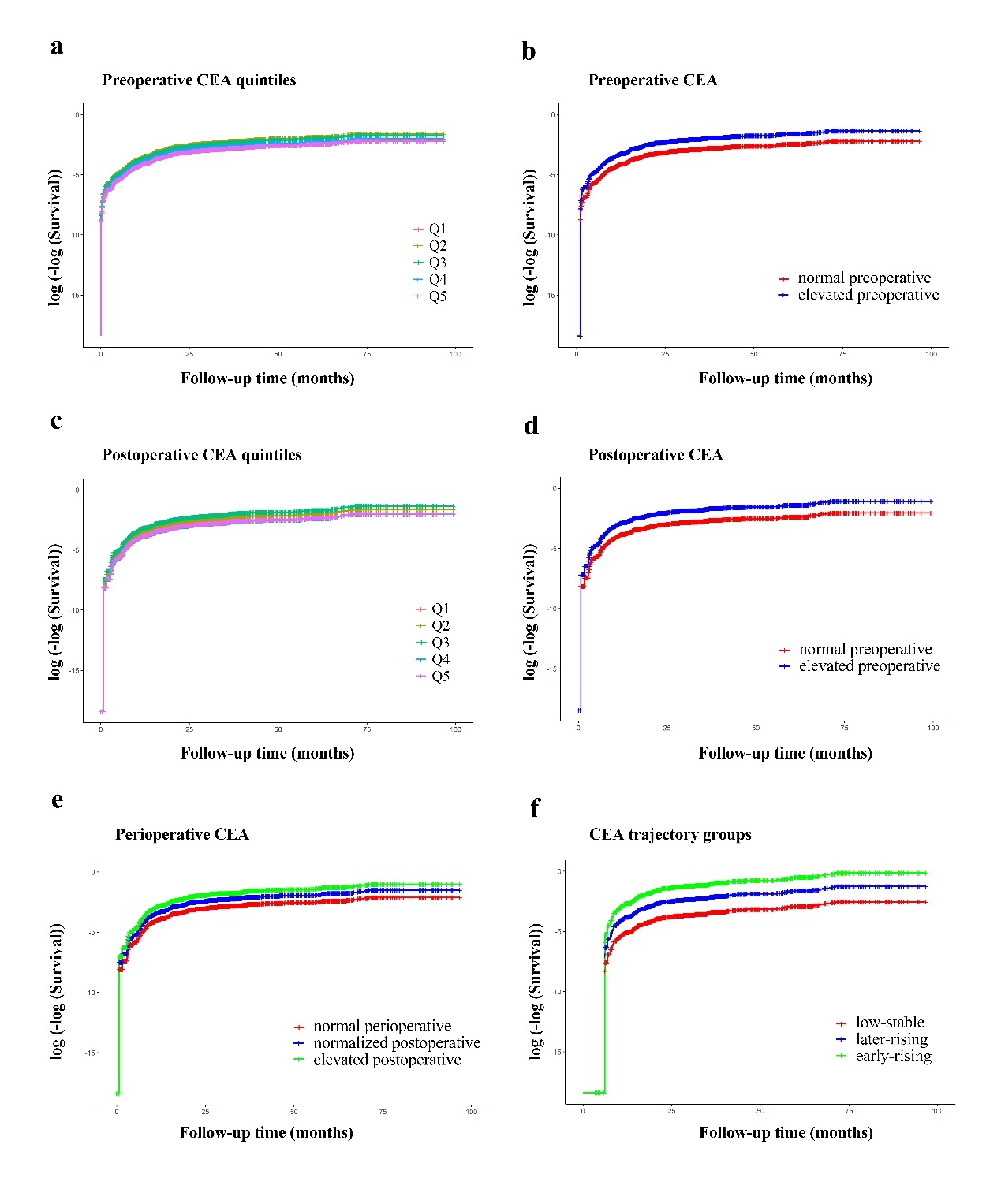


**Figure S3. Log–log plots of recurrence-free survival: (a–b) Preoperative CEA (quintiles, Q1–Q5, and dichotomized at 5 ng/mL); (c–d) Postoperative CEA (quintiles, Q1–Q5, and dichotomized at 5 ng/mL); (e) Perioperative CEA groups; (f) CEA trajectory groups.**


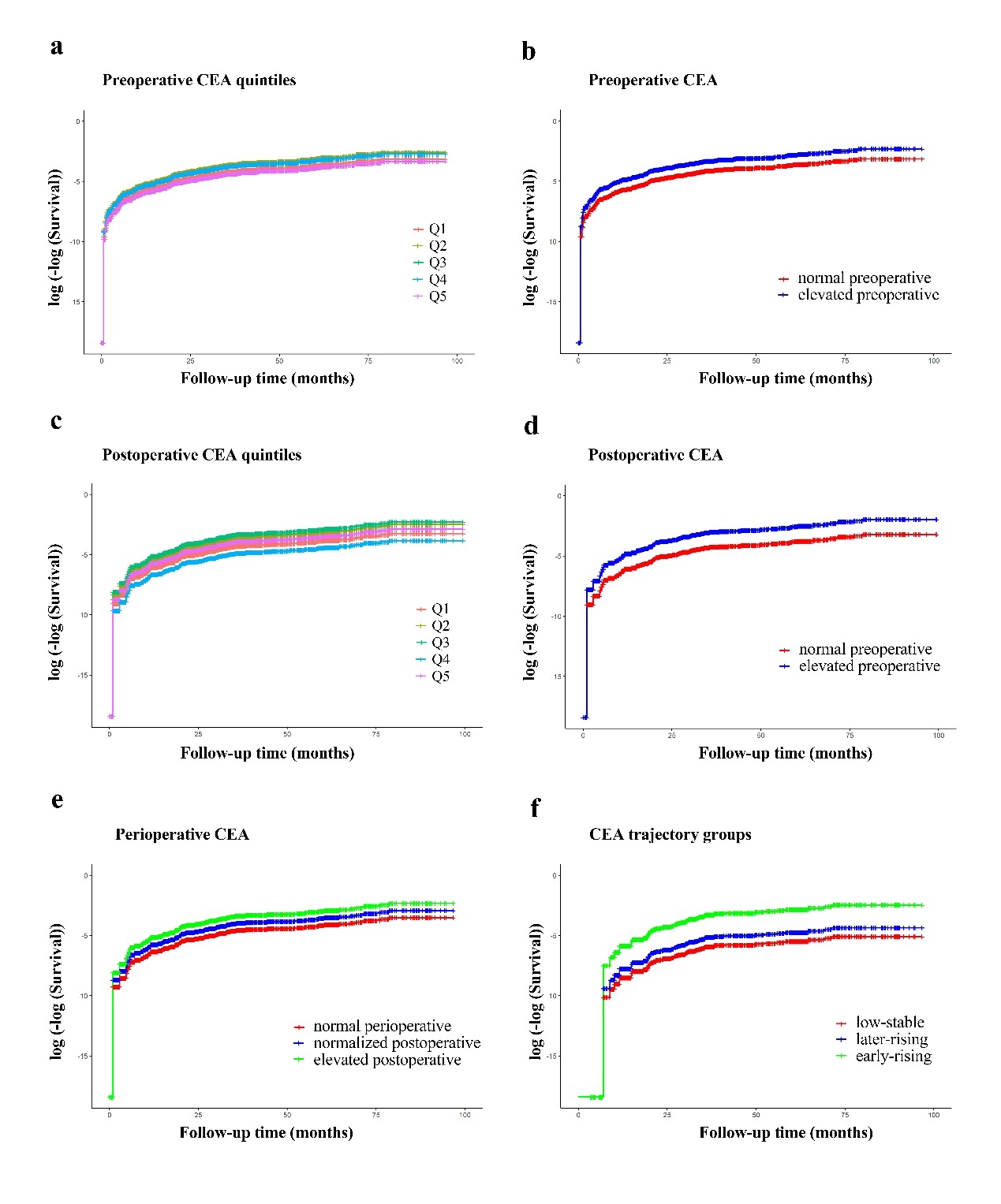


**Figure S4. Log–log plots of overall survival: (a–b) Preoperative CEA (quintiles, Q1–Q5, and dichotomized at 5 ng/mL); (c–d) Postoperative CEA (quintiles, Q1–Q5, and dichotomized at 5 ng/mL); (e) Perioperative CEA groups; (f) CEA trajectory groups.**


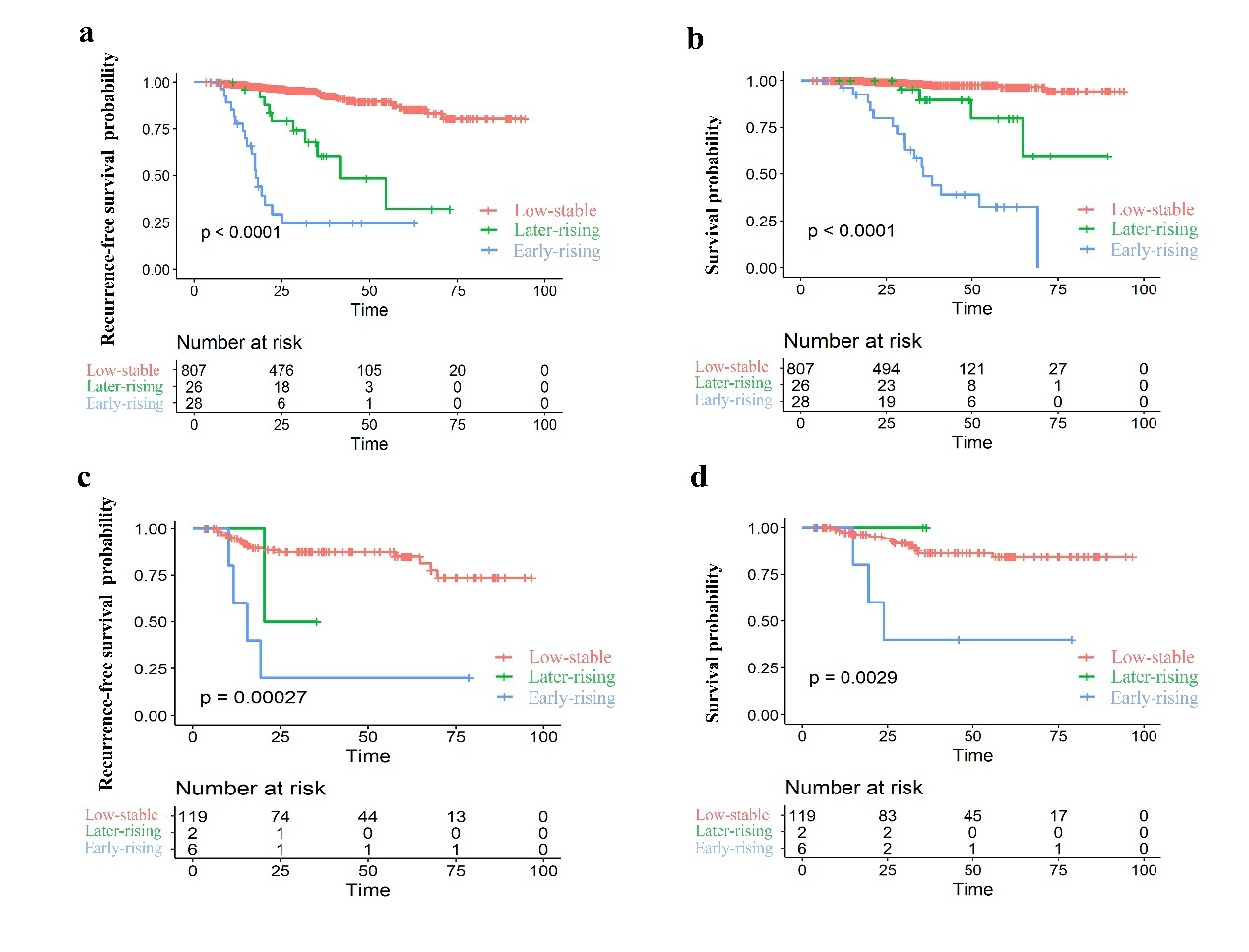


**Figure S5. Recurrence-free (left) and overall survival (right) by CEA trajectory groups, stratified by histology: (a–b) Adenocarcinoma; (c–d) Squamous cell carcinoma.**
